# Trace metals content in soils along the state road 51 (northeastern Poland)

**DOI:** 10.1007/s10661-013-3562-z

**Published:** 2014-01-07

**Authors:** Beata Modrzewska, Mirosław Wyszkowski

**Affiliations:** Department of Environmental Chemistry, University of Warmia and Mazury in Olsztyn, Plac Łódzki 4, 10-727 Olsztyn, Poland

**Keywords:** Road, Car traffic, Pollution, Soil, Trace elements

## Abstract

The aim of the study was to determine concentrations of some trace elements (lead, cadmium, chromium, and nickel) in soils along State Road 51 leading from Olsztyn to Olsztynek, northeastern Poland. The traffic flow had a significant effect on the content of heavy metals in soils lying along the road. Further away from the road, and under a lower traffic flow intensity, the amounts of contaminants originating from the motor traffic decreased. There was a pine forest growing by the road near Olsztyn, which served as a buffer zone for all the analyzed heavy metals. At all the sampling locations, the content of chromium was approximately the same as its natural concentration. The statistical analysis demonstrated that there was a strong negative correlation between the concentrations of nickel, lead, chromium, and cadmium in soils and the distance from the road. The biggest differences in the content of an individual element were determined for lead and the smallest ones—for cadmium. Emissions of trace elements depended on the differences in the traffic flow intensity, number, type, and speed of vehicles and on the atmospheric conditions as well as the distribution of buildings.

## Introduction

Soil is one of the most valuable nonrenewable resources, which—at the same time—remains among the most severely threatened elements of the natural environment (Massas et al. 2013). Soil degradation leads to persistent and usually irreversible changes. Soil is a mixture of a number of components, including organic matter, mineral constituents, all kinds of soil-borne organisms, water, air and man-made elements (Coskun et al. 2006; Faiz et al. 2009). Depending on the location, soil can also contain pollutants originating from industrial emissions, waste disposal sites, agriculture, urban centers, or from emissions generated by motor vehicles. Contaminants from all these sources are quickly transferred to air, water, and soil (Faiz et al. 2009; Liu et al. 2012) or to agricultural crops (Singh and Kumar 2006; Qiao et al. 2011). Contamination of the environment has risen drastically over the past century, which is an issue raised by Faiz et al. (2009) or Masses et al. (2013). The situation has been aggravated by the rapid development of our civilization, including urbanization and industrialization. On the one hand, heavy metals present in trace amounts are essential for maintaining proper functions of live organism (Zhang et al. 2009; Guala et al. 2013). They include zinc, copper, and manganese (D'Emilio et al. 2012; Massas et al. 2013). On the other hand, excessive quantities of these elements pose a threat to the human health and natural environment (Zhang et al. 2009; Duong and Lee 2011; Qiao et al. 2011; Liu et al. 2012; Massas et al. 2013; Sagi and Yigit 2012). Heavy metals accumulate rather than decompose in nature. When they exceed a certain threshold of their content in soil, a toxic effect on organisms is exerted (Martinez and Motto 2000; Ciećko et al. 2001; Wyszkowska and Wyszkowski 2002, 2003; Coskun et al. 2006; Wyszkowski and Wyszkowska 2006, 2009; Faiz et al. 2009; Duong and Lee 2011; Zhang et al. 2009; Massas et al. 2013). Higher emissions of heavy metals to the atmospheric air mean that heavy metals settle on roadside plants, possibly leading to their elevated concentrations. This is particularly threatening because plants growing on soil contaminated with trace elements accumulate large amounts of these constituents (Wyszkowski and Wyszkowska 2012; Wyszkowski and Radziemska 2013), which then, through the feeding chain, can enter into animal bodies and finally reach people's organisms, posing a health hazard (Coskun et al. 2006; Singh and Kumar 2006; Sagi and Yigit 2012; Guala et al. 2013). Trace elements appear in soil in a variety of forms, which differ in mobility and plant availability (D'Emilio et al. 2012; Guala et al. 2013). The mobility of elements is affected by the type of parent rock, the industrial and agricultural activities carried out in the environs (Zhang et al. 2009), type of soil, pH, soil sorption capacity, content of organic matter, and type of metal (Antoniadis and McKinley 2003; Ciećko et al. 2004, 2005, 2006; Guala et al. 2013), with the trace elements which originate from the parent rock demonstrating lower mobility than the same elements imported to soil from anthropogenic sources (Wilson and Bell 1996).

The number of motor vehicles has increased dramatically in the past years. This means constantly rising contamination of the soil environment with heavy metals. According to Christoforidis and Stamatis (2009), Faiz et al. (2009), Johansson et al. (2009), Helmreich et al. (2010), Duong and Lee (2011), Khan et al. (2011), and Liu et al. (2012), most of the heavy metals originate from exhaust fumes, oil leaks from cars, the wearing out of tires and brake disks and from corrosion of metal parts of vehicles. The level of soil contamination with heavy metals depends on the type of soil, climate, anthropogenic activity (Qiao et al. 2011), but also on atmospheric conditions (Duong and Lee 2011), including the direction and velocity of wind, kind of precipitation, land relief (Khan et al. 2011; Kluge and Wessolek 2012), or on the plant cover (Kluge and Wessolek 2012). Trace elements found in soil are also derived from the lithosphere, being released during soil formation processes and therefore constituting the natural background of their total concentration (Coskun et al. 2006; D'Emilio et al. 2012). Among the most significant pollutants which settle in soils along roads are heavy metals, including copper, chromium, cadmium, lead, zinc, manganese, nickel, and cobalt (Faiz et al. 2009). Most of the trace elements in soils along transportation routes appear in combination with other metals derived from the same anthropogenic source (Qiao et al. 2011), for example copper, zinc, and lead (Faiz et al. 2009; Zhang et al. 2009). In contrast, cadmium and nickel most often come from other sources (Zhang et al. 2009).

The above considerations encouraged this research, whose aim was to determine concentrations of some trace elements (lead, cadmium, chromium, and nickel) in soils along State Road 51 leading from Olsztyn to Olsztynek, northeastern Poland.

## Material and methods

### Collection of samples

**Fig. 1:**
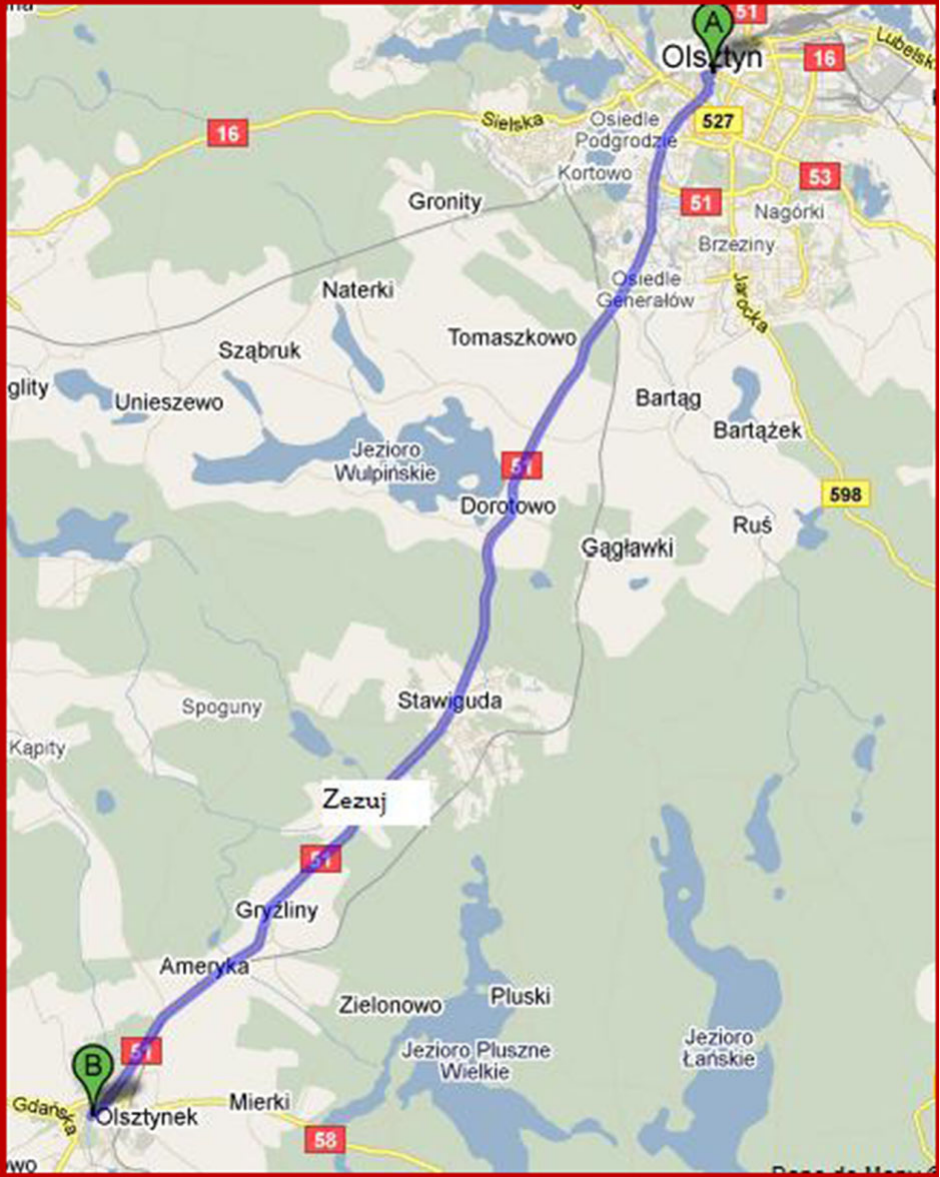
Map presenting the distribution of sampling sites (base map data from ©2013 Google)

Determinations of the content of trace metals were performed on samples of soils collected along State Road 51 leading from Olsztyn to Olsztynek (northeastern Poland). The traffic flow on this road is rather high, reaching an average of 12,581 vehicles a day, including 2,019 lorries and 105 coaches, along the road section from Olsztyn to Stawiguda; the section of the road from Stawiguda to Olsztynek carried 10,019 vehicles, including 2,051 lorries and 105 coaches (GDDKiA 2010). The traffic flow was measured by trained observers either manually or using automated techniques (video recording and traffic flow measurement posts). The soil material was collected from the surface layer at six sites located in the following localities: Olsztyn, Dorotowo, Stawiguda, Zezuj, Ameryka, and Olsztynek (Fig. 1). At each location, soil samples were taken from four sampling sites situated on the roadside and 15, 50, and 100 m off the road. Soil samples collected in Olsztyn originated from a coniferous forest with a pine stand (dominant type of woodland in Poland), whereas the other samples came from the sites overgrown with herbaceous plants, which in two locations, namely Zezuj and Ameryka, consisted of rudimentary plants.

### Analysis of samples

The soil material was dried at ambient temperature, ground, and passed through a 0.1 mm mesh sieve. Afterwards, the following determinations were made: total concentrations of four trace elements, i.e., lead, cadmium, chromium, and nickel, with the flame atomic absorption spectrometric method in an air–acetylene flame. First, the soil material was wet digested in nitric acid (HNO_3_ analytically pure) of the concentration equal 1.40 g·cm^−3^. The digestion was carried out in HP500 Teflon vessels placed in a MARS 5 microwave oven (CEM Corporation, USA). The process parameters, i.e., weight of analytical samples, volume of nitric acid, and temperature of the mineralization process were compliant with the protocol US-EPA3051 (1994). The results were processed statistically using a two-factorial analysis of variance ANOVA from Statistica (StatSoft, Inc. 2010). Pearson's simple correlation coefficients (*r*) were calculated between the differences.

## Results

**Fig. 2:**
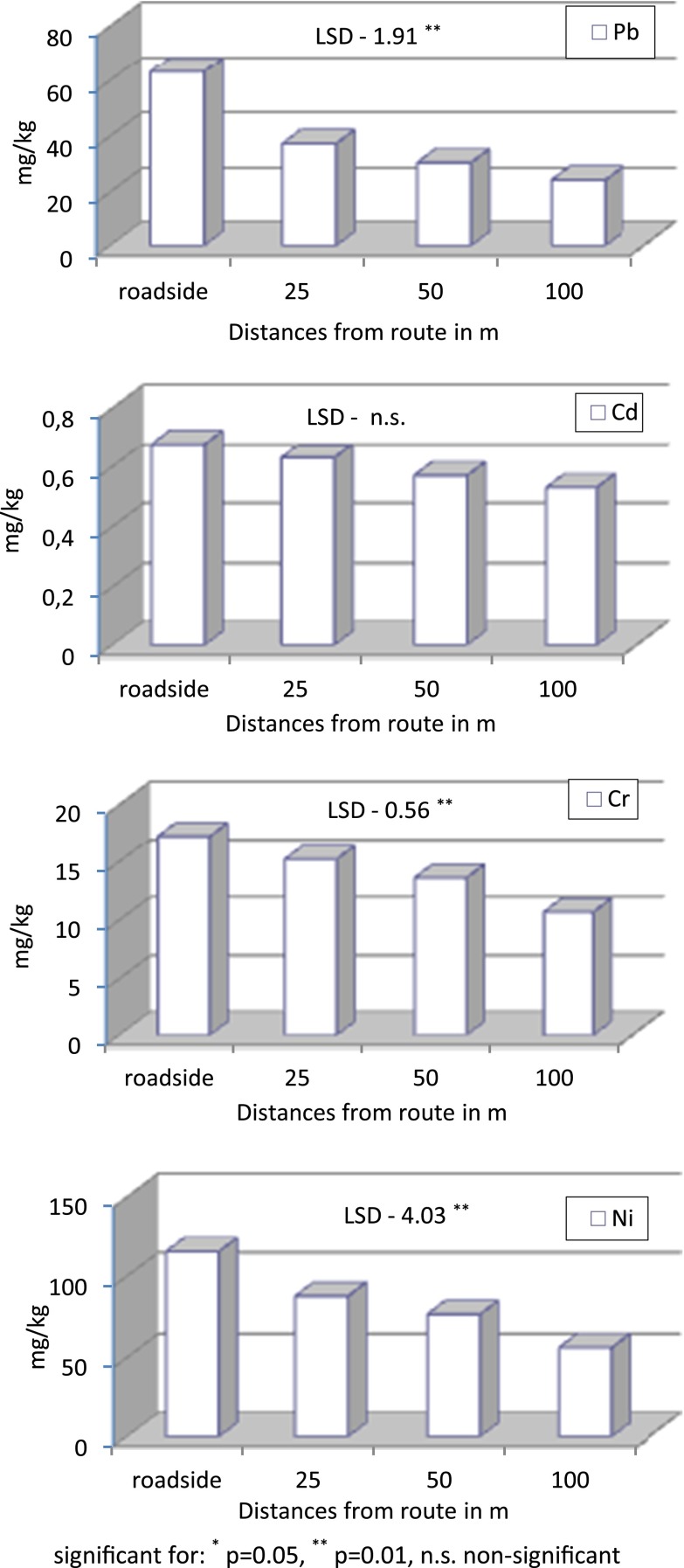
Content of trace elements in the surface soil layer near the State Road 51 from Olsztyn to Olsztynek—average from all soil points, in milligram per kilogram of soil

Concentrations of trace elements depended on the distance from the road and location (Tables 1, 2, and 3, Fig. 2).

**Table 1:** Content of lead (Pb) and cadmium (Cd) in the surface soil layer near the State Road 51 from Olsztyn to Olsztynek (in milligram per kilogram of soil)

Towns/villages	Distances from route in m				Average	*r*
	Along the roadside	25	50	100		
Lead (Pb)						
Olsztyn	52.7	17.8	15.0	11.6	24.3	−0.768^**^
Dorotowo	38.9	36.4	23.0	21.3	29.9	−0.900^**^
Stawiguda	96.9	23.2	19.6	18.6	39.6	−0.709^*^
Zezuj	52.5	32.1	21.2	18.9	31.2	−0.863^**^
Ameryka	53.7	36.1	32.6	28.4	37.7	−0.856^**^
Olsztynek	84.6	78.3	71.3	47.1	70.3	−0.988^**^
LSD for:	Town/village, 2.34^**^; distance from route, 1.91^**^; interaction, 4.68^**^					
Cadmium (Cd)						
Olsztyn	0.36	0.33	0.29	0.26	0.31	−0.975^**^
Dorotowo	0.53	0.49	0.40	0.36	0.44	−0.956^**^
Stawiguda	0.69	0.56	0.52	0.45	0.56	−0.936^**^
Zezuj	0.76	0.69	0.64	0.60	0.67	−0.953^**^
Ameryka	0.87	0.82	0.76	0.72	0.79	−0.967^**^
Olsztynek	0.90	0.86	0.84	0.80	0.85	−0.983^**^
LSD for:	Town/village, n.s.; distance from route, n.s.; interaction, n.s.					

*r* correlation coefficient, *n.s.* nonsignificant

^*^ *p* = 0.05; ^**^ *p* = 0.01

**Table 2:** Content of chromium (Cr) and nickel (Ni) in the surface soil layer near the State Road 51 from Olsztyn to Olsztynek (in milligram per kilogram of soil)

Towns/villages	Distances from route in m				Average	*r*
	Along the roadside	25	50	100		
Chromium (Cr)						
Olsztyn	14.2	13.1	10.8	1.4	9.9	−0.968^**^
Dorotowo	18.0	13.8	10.8	10.4	13.3	−0.872^**^
Stawiguda	19.2	17.8	16.9	14.6	17.1	−0.998^**^
Zezuj	20.2	18.6	17.0	16.5	18.1	−0.920^**^
Ameryka	17.7	15.7	14.6	13.2	15.3	−0.964^**^
Olsztynek	14.6	12.5	11.2	7.9	11.6	−0.997^**^
LSD for:	Town/village, 0.69^**^; distance from route, 0.56^**^; interaction, 1.38^**^					
Nickel (Ni)						
Olsztyn	109.4	66.2	52.9	41.5	67.5	−0.880^**^
Dorotowo	100.4	84.1	77.8	70.7	83.3	−0.924^**^
Stawiguda	95.6	73.3	56.9	30.7	64.1	−0.991^**^
Zezuj	99.3	74.9	57.3	36.2	66.9	−0.977^**^
Ameryka	133.1	94.9	91.2	87.9	101.8	−0.768^**^
Olsztynek	153.3	133.5	123.7	69.5	120.0	−0.987^**^
LSD for:	Town/village, 4.94^**^; distance from route, 4.03^**^; interaction 9.88^**^					

*r* Correlation coefficient, *n.s*. nonsignificant

^*^ *p* = 0.05; ^**^ *p* = 0.01

**Table 3:** Correlation coefficients (*r*) between lead, cadmium, chromium, and nickel and the content of other trace elements in the soils

	Cd	Cr	Ni	Mn	Zn	Cu	Fe
Pb	0.65^**^	0.25	0.80^**^	0.69^**^	0.85^**^	0.87^**^	0.75^**^
Cd		0.38^**^	0.65^**^	0.68^**^	0.57^**^	0.55^**^	0.65^**^
Cr			0.23	0.28	0.22	0.04	0.22
Ni				0.77^**^	0.82^**^	0.85^**^	0.94^**^

^**^ *p* = 0.01

### Lead

The average content of lead in the soils along the Olsztyn–Olsztynek road was from 24.3 to 70.3 mg/kg of soil (Table 1, Fig. 2). The Regulation of the Polish Minister for the Environment (2002) sets the permissible level of this trace element in the surface soil horizons at no more than 50 mg (protected areas), 100 mg (nonprotected areas, including arable lands) and 600 mg/kg of soil (land under transportation routes). The highest lead content was determined near the road in the village of Stawiguda, where it reached 96.9 mg/kg of soil, and in the town of Olsztynek, where it was 84.6 mg/kg of soil. Thus, the content of lead in the analyzed soils was below the allowable threshold. The lowest content of lead was determined in the soil in Dorotowo. The sampling sites in this village were situated between State Road 51 and an inner local road. The lead content on the roadside of State Road 51 was 38.9 mg/kg of soil, falling down to 21.3 mg/kg of soil behind the inner road. The location of Dorotowo could have had some influence on the little variation in the content of lead near and 100 m away from the road. By being a much larger town, Olsztyn should be characterized by the highest content of lead in soil. Contrary to the expectations, the soil sampled on the roadside in Olsztyn contained relatively little of this metal (52.7 mg/kg of soil), which may be attributed to the buffering function of the pine forest growing on that site. The data contained in Table 1 shows that the highest lead contamination of soil appeared in the belt situated in the immediate proximity of the road, decreasing stepwise further away from it. This is the proof that the road traffic and emission of fumes caused by cars have an impact on the soil content of lead. At a distance of 100 m from the road, the content of lead was on average two- to threefold lower than on the roadside. The biggest differences occurred in Olsztyn and Stawiguda, where fivefold less lead was determined 100 ms from the road than on the roadside. In short, the distance from the road had a significant effect on the content of lead in soil. At the other locations, the differences were smaller although highly significant.

### Cadmium

The average concentrations of cadmium in the analyzed soils ranged from 0.31 to 0.85 mg/kg of soil (Table 1, Fig. 2). The Regulation of the Polish Minister for the Environment (2002) specifies that the allowable amounts of cadmium are from 1 mg (protected areas), 4 mg (nonprotected areas, including farmland) up to 15 mg/kg of soil (lands under transportation routes). The highest cadmium concentration was determined in Olsztynek (0.90 mg/kg of soil) and Ameryka (0.87 mg/kg of soil); the lowest cadmium content in soil occurred in Olsztyn (0.36 mg/kg of soil). The overall results suggest that the distance from the road and the influence of traffic were less important factors affecting the content of cadmium in soil than that of the other analyzed heavy metals. However, the content of cadmium decreased steadily further away from the road at all the sampling sites. At a distance of 100 m from the road, the concentration of cadmium was less by 11 % in Olsztynek, 28 % in Olsztyn, 32 % in Dorotowo, and 35 % in Stawiguda compared to the determinations made in soil samples obtained from the roadside. With respect to the aforementioned Regulation (2002), the concentrations of cadmium in the analyzed soils were below the allowable limits.

### Chromium

The average content of chromium in soil ranged from 9.9 mg/kg in Olsztyn to 18.1 mg/kg of soil in Zezuj (Table 2, Fig. 2). The Regulation of the Polish Minister for the Environment (2002) established the maximum allowable limit of chromium at 50 mg on protected land, 150 mg on nonprotected land, including farmland, and 500 mg/kg of soil under transportation routes. The highest level of chromium was determined in the soil sampled from the roadside in Zezuj (20.2 mg/kg) and in Stawiguda (19.2 mg/kg of soil). The lowest content of chromium in soil was determined in the roadside in Olsztyn and in Olsztynek (14.2 and 14.6 mg/kg of soil, respectively). The distance from the road had a considerable influence on the content of this element. This effect was particularly notable in Olsztyn, where tenfold less chromium was detected at a distance of 100 m than on the roadside. At other locations, the differences were smaller, ranging from 18 % (Zezuj) to 42 % (Dorotowo) and 46 % (Olsztynek). By analyzing the data in Table 3, it could be concluded that chromium in those soils appeared in the natural concentrations and that the permissible level was not exceeded at any of the locations, not even in the soils lying directly near the road.

### Nickel

The average content of nickel in the soils along the Olsztyn–Olsztynek route was within 64.1–120.0 mg/kg of soil (Table 2, Fig. 2). The Regulation of the Polish Minister for the Environment (2002) states that the allowable content of this metal is 35 mg (protected areas), 100 mg (nonprotected areas, including arable land, and 300 mg/kg of soil (under transportation routes). The highest content of nickel was determined on the roadside near Olsztynek (153.3 mg/kg) and in Ameryka (133.1 mg/kg of soil). Both localities lie near State Road 7, which proves that the detected contamination has an anthropogenic origin. The sites where the samples were taken had a significant effect on the results because 100 m away from the road the concentration of nickel was from 1.4-fold (in Dorotowo) to 3.1-fold (Stawiguda) lower than in the nearest proximity of the road. With respect to the above Regulation (2002), the content of nickel determined in the analyzed soils was below the maximum allowable limit.

The calculated correlation coefficients (*r*) indicate significant relationships between lead, cadmium, and nickel versus the content of other trace elements in the soils (Table 3). However, no significant relationships were detected between chromium and the other micro-elements.

## Discussion

The concentrations of the trace elements were the highest in soils lying next to the road, but decreased at further distances. A similar dependence was observed by Kluge and Wessolek (2012). This observation suggests that soils near roads are contaminated with metals from anthropogenic sources, e.g., from motor transport in our study, as there are not any larger industrial plants near the road from Olsztyn to Olsztynek. Duong and Lee (2011) claimed that the velocity and intensity of traffic flow had a strong influence on the degree of soil contamination with heavy metals. The observations reported by Faiz et al. (2009) as well as Duong and Lee (2011) imply that the number of motor vehicles has increased in the recent years. Higher speeds reached by cars mean that both tires and road surfaces are worn out faster, which adds to a higher content of elements in roadside soil. According to Helmreich et al. (2010), apart from the traffic flow rate, another factor which affects the accumulation of contaminants in soils near roads is the weather, namely prolonged periods of drought as well as the duration and intensity of precipitations. All the above factors may have contributed to the elevated concentrations of the trace elements detected along the Olsztyn–Olsztynek road. Faiz et al. (2009), Johansson et al. (2009), Helmreich et al. (2010), Duong and Lee (2011), and Liu et al. (2012) state that emissions from the road traffic have a significant effect on soil contamination with nickel, lead, and cadmium along streets. They verified that one of the reasons was the wearing out of brakes and tires due to frequently stopping a car, a phenomenon which was also responsible for an increased emission of exhaust fumes. Moreover, chromium and nickel are found in various metal alloys, which are exposed to corrosion and release the said elements back to the environment (Christoforidis and Stamatis 2009). Duong and Lee (2011) added that during the intensive exploitation of motor vehicles coinciding with strong winds, particles from cars could travel over considerable distances. The results presented hereinabove provide grounds for a conclusion that the highest concentrations of nickel, lead, and cadmium were near the road, which carried a large number of vehicles moving at a high speed.

The observations reported by Helmreich et al. (2010) seem to prove that a high content of one of the elements results from an equally high content of another element, which corroborates the conclusion that both contents are interrelated and originate from the same source (Qiao et al. 2011). These authors claim that lead and cadmium are typical anthropogenic contaminants, and have a common source on roads, such as the wearing out of tires and brake disks, as well as oil or petrol leaks (Faiz et al. 2009; Khan et al. 2011). In a study reported by Faiz et al. (2009), the concentration of cadmium in soils was the lowest compared to the other analyzed elements. Similar results were obtained in the present investigation, because cadmium—according to Helmreich et al. (2010)—is present in trace amounts in tires and brake disks of cars. Helmreich et al. (2010) added that another source of nickel and cadmium contamination of soils near roads is the wearing out of the road surface and use of paints containing lead. Other researchers, for example Johansson et al. (2009) or Khan et al. (2011), claim that both lead and nickel come from exhaust fumes. Among the elements discussed in this paper, lead makes a noteworthy case. Several researchers, e.g., Helmreich et al. (2010) and D'Emilio et al. (2012), have proven that the gradual replacement of leaded with unleaded petrol has had a positive effect on soil contamination with this element. The cited authors observed that the content of lead in the soil environment had been decreasing. The same tendency is evident in this study, too, because the content of lead decreased steadily as we moved further from the road.

The availability and mobility of each trace element depends on a number of physicochemical factors, of which pH seems most important (Singh and Kumar 2006). Wilson and Bell (1996) state that forest soils are more acidic in reaction than arable lands, which may stimulate higher concentrations of heavy metals. Cadmium, chromium, cobalt, copper, nickel, lead, manganese, and zinc have been classified as the heavy metals most readily dissolved in acid environment. Cadmium is the most mobile metal in the soil environment, in contrast to less mobile lead. However, all trace metals become more mobile when the soil reaction turns more acidic (Wilson and Bell 1996). While analyzing the content of heavy metals in forest soils near Olsztyn, it becomes apparent that the woodland cover of these soils did not stimulate higher levels of the discussed elements. The reason could be the buffering function of the forest.

Guala et al. (2013) concludes that the mobility of trace elements in soil depends on changes in the soil pH. An increase in the soil acidity causes an increase in the solubility of the discussed heavy metals (Knight et al. 1998; Ciećko et al. 2001). According to Martinez and Motto (2000), Singh and Kumar (2006), Kluge and Wessolek (2012), and Massas et al. (2013), other soil properties which influence the solubility and bioavailability of heavy metals are the content of organic matter, soil sorption capacity, and soil texture. The quoted scholars observed that soils characterized by a low sorption capacity and a low content of organic matter as well as loose and sandy soils most probably contained higher quantities of heavy metals. According to Singh and Kumar (2006), organic matter is able to bind trace elements and therefore limits their availability to plants. Another significant factor is also the actual content of a given element in soil. The present results suggest that soil more distant to the road had more organic matter and a slightly acid or slightly alkaline pH, which successfully prevented the toxic effect of trace metals on plants.

The concentrations of nickel, lead, and cadmium in the immediate proximity of the road were higher in Stawiguda than in the other localities. This was most probably a result of the more compact arrangement of detached houses, heated with fossil fuels. Despite the numerous sources of pollution, which may affect the load of soil with heavy metals, it is justifiable to conclude that most of these contaminants near roads are derived from the exploitation of motor vehicles and road surfaces. Furthermore, the sites used for sampling soil in the present research were located near a road which carried a heavy load of vehicles.

## Conclusions

The traffic flow had a significant effect on the content of heavy metals in soils lying along the road. Further away from the road and under lower traffic flow intensity, the amounts of contaminants originating from the motor traffic decreased. There was a pine forest growing by the road near Olsztyn, which served as a buffer zone for all the analyzed heavy metals. At all the sampling locations, the content of chromium was approximately the same as its natural concentration. The statistical analysis demonstrated that there was a strong negative correlation between the concentrations of nickel, lead, chromium, and cadmium in soils and the distance from the road. The biggest differences in the content of an individual element were determined for lead and the smallest ones—for cadmium. The proximity of a large town as well as a high intensity of the traffic flow was responsible for an elevated concentration of chromium. Emissions of trace elements depended on the differences in the traffic flow intensity, number, type, and speed of vehicles and on the atmospheric conditions as well as the distribution of buildings. In recent years, the number of motor vehicles has increased and so has the content of roadside soils. The Province of Warmia and Mazury is characterized by soils with a low abundance of trace metals and only point excess of such pollutants, due to the concentration of traffic flow, local heat, and power generator plants and—to a much lesser extent—because of the agricultural production on soils. The largest areas with soils containing raised amounts of heavy metals lie in the south and southwestern parts of Poland.
